# Pre-hospital transdermal glyceryl trinitrate in patients with stroke mimics: data from the RIGHT-2 randomised-controlled ambulance trial

**DOI:** 10.1186/s12873-021-00560-x

**Published:** 2022-01-10

**Authors:** Bronwyn Tunnage, Lisa J. Woodhouse, Mark Dixon, Craig Anderson, Sandeep Ankolekar, Jason Appleton, Lesley Cala, Timothy England, Kailash Krishnan, Diane Havard, Grant Mair, Keith Muir, Steve Phillips, John Potter, Christopher Price, Marc Randall, Thompson G. Robinson, Christine Roffe, Else Sandset, Niro Siriwardena, Polly Scutt, Joanna M. Wardlaw, Nikola Sprigg, Philip M. Bath, on behalf of the RIGHT-2 Investigators

**Affiliations:** 1 Stroke Trials Unit, Division of Clinical Neuroscience, University of Nottingham, Nottingham, NG5 1PB UK; 2 Department of Paramedicine, Auckland University of Technology, Auckland, New Zealand; 3 East Midlands Ambulance Service NHS Trust, Nottingham, NG8 6PY UK; 4 The George Institute for Global Health, Faculty of Medicine, University of New South Wales, New South Wales, Australia; 5 The George Institute China at Peking University Health Science Center, Beijing, China; 6 Neurology Department, Royal Prince Alfred Hospital, Sydney Health Partners, Sydney, NSW Australia; 7 Department of Neurology, King’s College Hospital NHS Foundation Trust, London, UK; 8 Neurology, University Hospitals Birmingham NHS Foundation Trust, Birmingham, UK; 9 Faculty of Health and Medical Sciences, University of Western Australia, Perth, Australia; 10 Vascular Medicine, Division of Medical Sciences and Graduate Entry Medicine, University of Nottingham, Royal Derby Hospital Centre, Derby, UK; 11 Stroke, Nottingham University Hospitals NHS Trust, Nottingham, UK; 12 Centre for Clinical Brain Sciences, Edinburgh Imaging and UK Dementia Research Institute at the University of Edinburgh, Chancellor’s Building, Edinburgh, UK; 13 University of Glasgow, Queen Elizabeth University Hospital, Glasgow, UK; 14 Department of Medicine, Dalhousie University and Queen Elizabeth II Health Sciences Centre, Halifax, NS B3H 3A7 Canada; 15 Bob Champion Research and Education Building, University of East Anglia, Norwich, UK; 16 Institute of Neuroscience, Newcastle University, Newcastle, UK; 17 Department of Neurology, Leeds Teaching Hospitals NHS Trust, Leeds, UK; 18 Department of Cardiovascular Sciences and NIHR Leicester Biomedical Research Centre, University of Leicester, Leicester, UK; 19 Stroke Research in Stoke, Institute for Science and Technology in Medicine, Keele University, Stoke-on-Trent, UK; 20 Department of Neurology, Oslo University Hospital, Oslo, Norway; 21 Research and Development, The Norwegian Air Ambulance Foundation, Oslo, Norway; 22 Community and Health Research Unit, University of Lincoln, Lincoln, UK; 23 Hearing Sciences, NIHR Nottingham Biomedical Research Centre, Nottingham, UK

**Keywords:** Stroke, Mimic, Functional stroke, Migraine, Seizures, Glyceryl trinitrate, Nitroglycerin, Ambulance, Paramedic

## Abstract

**Background:**

Prehospital stroke trials will inevitably recruit patients with non-stroke conditions, so called stroke mimics. We undertook a pre-specified analysis to determine outcomes in patients with mimics in the second Rapid Intervention with Glyceryl trinitrate in Hypertensive stroke Trial (RIGHT-2).

**Methods:**

RIGHT-2 was a prospective, multicentre, paramedic-delivered, ambulance-based, sham-controlled, participant-and outcome-blinded, randomised-controlled trial of transdermal glyceryl trinitrate (GTN) in adults with ultra-acute presumed stroke in the UK. Final diagnosis (intracerebral haemorrhage, ischaemic stroke, transient ischaemic attack, mimic) was determined by the hospital investigator. This pre-specified subgroup analysis assessed the safety and efficacy of transdermal GTN (5 mg daily for 4 days) versus sham patch among stroke mimic patients. The primary outcome was the 7-level modified Rankin Scale (mRS) at 90 days.

**Results:**

Among 1149 participants in RIGHT-2, 297 (26%) had a final diagnosis of mimic (GTN 134, sham 163). The mimic group were younger, mean age 67 (SD: 18) vs 75 (SD: 13) years, had a longer interval from symptom onset to randomisation, median 75 [95% CI: 47,126] vs 70 [95% CI:45,108] minutes, less atrial fibrillation and a lower systolic blood pressure and Face-Arm-Speech-Time tool score than the stroke group. The three most common mimic diagnoses were seizure (17%), migraine or primary headache disorder (17%) and functional disorders (14%). At 90 days, the GTN group had a better mRS score as compared to the sham group (adjusted common odds ratio 0.54; 95% confidence intervals 0.34, 0.85; *p* = 0.008), a difference that persisted at 365 days. There was no difference in the proportion of patients who died in hospital, were discharged to a residential care facility, or suffered a serious adverse event.

**Conclusions:**

One-quarter of patients suspected by paramedics to have an ultra-acute stroke were subsequently diagnosed with a non-stroke condition. GTN was associated with unexplained improved functional outcome observed at 90 days and one year, a finding that may represent an undetected baseline imbalance, chance, or real efficacy. GTN was not associated with harm.

**Trial registration:**

This trial is registered with International Standard Randomised Controlled Trials Number ISRCTN 26986053.

**Supplementary Information:**

The online version contains supplementary material available at 10.1186/s12873-021-00560-x.

## Background

Glyceryl trinitrate (GTN) has several effects that may be beneficial in acute stroke. High blood pressure (BP) is common in the acute phase of stroke and associated with poor outcome [1]. In-hospital BP lowering is recommended for patients with intracerebral haemorrhage [2], and the application of a glyceryl trinitrate (GTN) skin patch is a simple and efficient approach. GTN has other effects which may be beneficial in stroke such as topping up nitrate-depleted endothelium.

Stroke can be difficult to diagnose in the prehospital setting as there is no perfect or readily available diagnostic test. Conditions such as seizures, migraine and functional disorders can present with symptoms suggestive of a stroke, hence use of the term ‘stroke mimics’ [3]. Mimics are estimated to account for 31% of presentations at hospital with suspected stroke [4]. An incorrect working diagnosis of stroke may delay appropriate treatment for patients and expose them to unnecessary risk as some may receive stroke treatments such as thrombolysis before the correct diagnosis is confirmed. Equally, patients with a stroke may be deprived of life-changing treatment if their initial diagnosis is thought to be a mimic. Numerous factors have been reported to be associated with a greater probability of an event being a mimic rather than stroke, including younger age, female sex, fewer vascular risk factors, history of seizures and less severe presenting symptoms including a lower likelihood of facial or limb weakness, speech difficulty or acute hypertension [5, 6]. The Face-Arms-Speech-Time (FAST) tool is widely used by ambulance paramedics to diagnose suspected stroke, and has a sensitivity of 79% [7]. However, as it is limited to examining the patient for facial palsy, altered motor functioning of the arm and abnormal speech, FAST is less likely to identify mild or severe strokes and those affecting only the posterior circulation [8, 9].

The second Rapid Intervention with Glyceryl trinitrate in Hypertensive stroke Trial-2 (RIGHT-2) investigated the effects of ultra-acute administration of transdermal GTN versus sham patch by paramedics in 1149 patients with suspected stroke in the UK [10]. The primary outcome was the 7-level modified Rankin Scale (mRS) score at 90 days. The RIGHT-2 trial tested the null hypothesis that GTN will not shift the mRS in participants with ultra-acute stroke. The alternative hypothesis, that GTN will shift the mRS between those stroke participants randomised to GTN versus sham, was 2-sided. Overall, no difference in the mRS was observed between the groups in participants with a final diagnosis of stroke or transient ischaemic attack. However, among the 297 (26%) participants with a final hospital diagnosis of a non-stroke condition, mRS scores were better in those randomised to GTN compared to sham [10]. The aim of this pre-specified subgroup analysis was to characterise stroke mimic cases in a FAST positive population and to examine in detail the RIGHT-2 primary outcome findings among participants with a stroke mimic as their final diagnosis.

## Methods

### Study design and population

RIGHT-2 was a multicentre, prospective, randomised, sham-controlled, participant-and-outcome-blinded, randomised-controlled trial in adults with ultra-acute presumed stroke in the UK. Paramedics from eight UK ambulance services (East Midlands, East of England, London, South Central, South West, Wales, West Midlands and Yorkshire) delivered the trial within the pre-hospital ambulance environment [10, 11]. Briefly, adult patients were eligible for inclusion if they accessed care through an emergency ambulance telephone call for presumed stroke and were assessed within 4 h of onset of their symptoms by a trial-trained paramedic from a participating ambulance service and could be transported to a participating hospital. Patients had to have at least two positive signs in the FAST test assessment (the number of positive signs was scored as a value out of a maximum of 3) and a systolic blood pressure (SBP) ≥ 120 mmHg. Patients from a nursing home, or with reduced consciousness (Glasgow coma scale [GCS] < 8/15), hypoglycaemia (capillary glucose < 2.5 mmol/l) or a witnessed seizure, were excluded. A sample size calculation determined that a total sample size of 850 participants (425 in each group) was required to detect a shift in mRS with a common odds ratio [OR] of 0·70 assuming an overall significance level of 5%, 90% power, 3% loss to follow-up, mimic and transient ischaemic attack rate of 20%, and reduction for baseline covariate adjustment of 20%. However, during the trial, the non-stroke diagnosis rate exceeded 30% and so the overall sample size was increased to 1050 to maintain the overall effect size and statistical power [10]. Detailed inclusion and exclusion criteria and additional information on the methods are given in the published protocol paper and in the Supplement to the main trial publication [11, 12].

The final diagnosis was made by the principal investigator based on clinical and neuroimaging findings, and categorised as intracerebral haemorrhage, ischaemic stroke, transient ischaemic attack (TIA) or stroke/TIA mimic. Brain scans were reviewed centrally by an expert panel of neuroradiologists who were aware of the time to scan and the side of symptoms but blinded to all other information to confirm diagnosis of ischaemic, haemorrhagic stroke or mimic with structural lesion. Diagnostic adjudication was completed without knowledge of the primary outcome. Diagnosis of stroke mimic was made from the final diagnosis recorded in the patient notes. Patients who had a stroke mimic received the same follow-up at Day 365 as confirmed stroke and TIA cases.

### Treatment

Participants were randomly assigned to receive transdermal GTN (nitroglycerin; 5 mg as Transiderm-Nitro® 5, Novartis, Frimley UK) or a similarly-appearing sham treatment not known to exert any pharmacological effect (DuoDERM® hydrocolloid dressing, Convatec, Flintshire UK) in a 1:1 ratio. Randomisation was stratified by ambulance station with blocks of four packs (two active, two control) in a random permuted order that was generated by the trial programmer at the Nottingham Stroke Trials Unit. Ambulances carried only one pack at a time and paramedics signed-out the treatment pack with the lowest randomisation number from their ambulance station at the start of their shift and returned it if unused at the end of their shift [10]. The first treatment (GTN or sham) was administered by the paramedic immediately after randomisation in the ambulance; further treatments were given daily for up to three days in hospital but were stopped earlier if a non-stroke diagnosis was made. Each treatment pack was sealed to maintain blinding of paramedics. Participants were effectively masked since the patches and dressings themselves were unlabelled, and a gauze dressing was taped over the top of the patch or dressing to provide additional masking.

### Outcomes

The primary outcome was functional status assessed across the 7-levels of the mRS (0 = no dependency to 6 = death) [13], measured at 90 days after randomisation. A trained assessor, masked to treatment allocation and using a structured questionnaire, obtained outcomes during a telephone interview with the participant. In cases where the participant was incapable of providing this information, the relative or carer was interviewed. If telephone contact could not be made after multiple attempts, a questionnaire was sent by post.

Participants were seen at Day 4 (or at hospital discharge, if earlier) to determine adherence to treatment and assess neurological deterioration. Duration of stay and discharge destination (to another hospital, institution or home) were also recorded. Pre-specified secondary clinical outcomes at Day 90 included activities of daily living (Barthel Index [BI]); cognition (modified telephone mini-mental state examination [MMSE]; telephone interview for cognition scale-modified [TICS-M]; and categorical verbal fluency using animal naming); health-related quality of life on the European quality of life-5 dimensions-3 level [EQ-5D-3L], from which a health status utility value [HSUV] was calculated with an EQ-visual analogue scale; and mood (abbreviated Zung depression score [ZDS]), all as used in the preceding Efficacy of Nitric Oxide in Stroke (ENOS) trial of GTN in hospital [10, 14]. Home-time was calculated as the number of days between discharge and Day 90. As a secondary assessment time, clinical outcomes were re-obtained by telephone at one year.

### Statistical analysis

Analyses followed the RIGHT-2 statistical analysis plan [15]. The primary outcome (shift on 7-level mRS) was analysed using ordinal logistic regression with adjustment for age, sex, pre-morbid mRS, baseline FAST score and SBP, and time from the onset of symptoms to randomisation. The assumption of proportional odds was tested using the likelihood ratio test. We also performed unadjusted, per-protocol and imputed (missing mRS data estimated using multiple regression-based imputation) sensitivity analyses for completeness. Heterogeneity of the treatment effect on the primary outcome was assessed for the purpose of hypothesis-generation in pre-specified subgroups by adding an interaction term to an adjusted ordinal logistic regression model. Death was analysed using adjusted Cox proportional hazards regression models. Other outcomes were assessed using adjusted binary logistic regression, Cox regression, ordinal logistic regression, multiple linear regression and analysis of covariance (BP). A pre-specified global outcome (comprising ordered categorical or continuous data for mRS, BI, ZDS, TICS-M and EQ-5D-HSUV) was analysed using the Wei-Lachin test [16]. Data are shown as number (%), median [interquartile range, IQR], mean (standard deviation, SD), difference in mean and odds ratio, with 95% confidence intervals (CI).

## Results

### Demographics

**Fig. 1:**
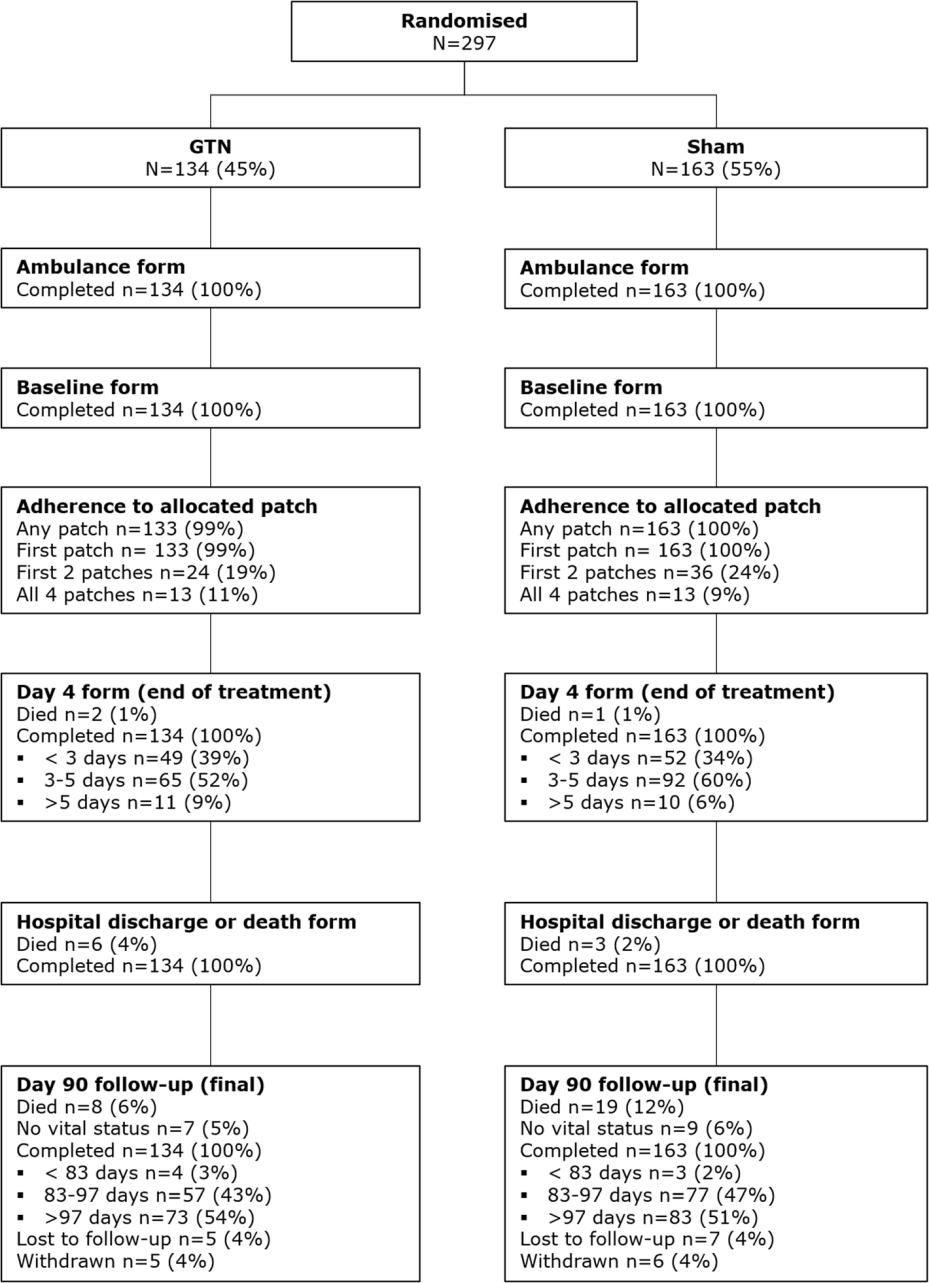
Trial profile for mimic group

From October 2015 to May 2018, 516 trial-trained paramedics enrolled 1149 participants into RIGHT-2 with follow-up continuing to 365 days. Among these 1149 patients, 297 (26%) were subsequently diagnosed with a stroke mimic (Fig. 1). Compared to stroke cases and prior to randomisation, the mimic group were on average significantly younger, had a longer interval from symptom onset to randomisation, a lower proportion of atrial fibrillation/flutter, lower SBP, and fewer positive signs in their FAST assessment (Additional Table A).

Among the 297 patients with a stroke mimic, the mean age was 67 years (SD 18), 53% of participants were female, and 13% were non-white (Table 1). The GCS score was less than 14 in one quarter of mimic cases, and less than half were positive on all three FAST indicators (face/arm/speech). In the mimic group, the most common pre-existing medical conditions were hypertension (51%), previous stroke (31%), diabetes mellitus (21%) and heart disease (21%). Twenty-eight percent of participants in the study had a pre-existing dependence of a moderate or greater severity (mRS > 2). The median time from symptom onset to randomisation was 75 min [IQR 47, 126].

**Table 1:** Baseline ambulance and hospital admission characteristics of Mimic patients enrolled in the RIGHT-2 trial. Data are number (%), median [IQR], or mean (standard deviation)

	All	GTN	Sham	Difference	2p
**Ambulance data (pre-randomisation)**					
Number of patients	297	134	163		
Age (years)	67 (18)	68 (19)	66 (18)	1.7 (− 2.4, 5.9)	0.41
< 60 (%)	107 (36)	49 (37)	58 (36)		
60–70 (%)	45 (15)	17 (13)	28 (17)		
70–80 (%)	55 (19)	22 (16)	33 (20)		
≥ 80 (%)	90 (30)	46 (34)	44 (27)		
Sex (female) (%)	157 (53)	74 (55)	83 (51)	4.3 (−7.1, 15.7)	0.46
Time from onset to randomisation (minutes)	75 [47,126]	72.5 [48,120]	79 [46,140]	−6.0 (−19.0, 5.0)	0.27
ECG, AF/flutter (%)	29 (13)	11 (11)	18 (15)	3.6 (−5.1, 12.3)	0.42
Systolic blood pressure (mmHg)	159 (26)	155 (24)	162 (27)	−7.0 (−12.9, − 1.1)	**0.021**
Diastolic blood pressure (mmHg)	91 (16)	89 (16)	92 (16)	−2.7 (−6.4, 1.0)	0.15
Heart rate (bpm)	83 (19)	82 (16)	84 (21)	−1.7 (−5.9, 2.6)	0.44
Glasgow coma scale	14 (2)	14 (2)	14 (2)	−0.3 (−0.7, 0.1)	0.13
Glasgow coma scale < 14 (%)	73 (25)	39 (29)	34 (21)	−8.2 (−18.2, 1.8)	0.11
FAST score (/3)	2 (1)	2 (1)	2 (1)	0.0 (−0.1, 0.2)	0.61
FAST score = 3 (%)	144 (49)	67 (50)	77 (48)	−2.9 (−14.3, 8.6)	0.63
**Hospital admission data (post randomisation)**					
Number of patients with data	297	134	163		
Ethnic group, non-white (%)	35 (13)	15 (12)	20 (13)	1.2 (−6.7, 9.0)	0.77
Ethnicity, White (%)	242 (87)	110 (88)	132 (87)		
Ethnicity, Black (%)	14 (5)	5 (4)	9 (6)		
Ethnicity, Asian (%)	18 (6)	10 (8)	8 (5)		
Ethnicity, Other (%)	3 (1)	0 (0)	3 (2)		
Pre-morbid mRS [/5]	1 [0,3]	1 [0,3]	0 [0,3]	0.00 (0.00, 0.00)	0.21
Pre-morbid mRS > 2 (%)	79 (28)	39 (31)	40 (26)	−4.7 (−15.3, 5.9)	0.38
Medical history (%)					
Hypertension	142 (51)	61 (49)	81 (53)	4.1 (−7.8, 15.9)	0.50
Diabetes mellitus	59 (21)	27 (22)	32 (21)	−0.6 (−10.3, 9.2)	0.91
Previous stroke	85 (31)	37 (30)	48 (32)	2.2 (−8.7, 13.1)	0.70
Ischaemic heart disease	58 (21)	29 (24)	29 (19)	−4.2 (−14.1, 5.6)	0.39
Smoking, current	54 (24)	26 (25)	28 (23)	−2.2 (−13.4, 8.9)	0.69
Antiplatelets	69 (36)	36 (40)	33 (33)	−7.0 (−20.7, 6.7)	0.32
Anticoagulants	33 (17)	15 (17)	18 (18)	1.0 (−9.7, 11.7)	0.86
Either	96 (51)	47 (52)	49 (49)	−3.2 (−17.5, 11.0)	0.66
OCSP syndrome, TACS (%)	40 (18)	15 (16)	25 (19)	3.6 (−6.4, 13.6)	0.49
NIHSS (/42)	4 [2,8]	4 [1,9]	4 [2,7]	0.0 (−1.0, 1.0)	0.64

*AF* atrial fibrillation, *bpm* beats per minute, *ECG* electrocardiogram, *FAST* face-arm-speech test, *GTN* glyceryl trinitrate, *IQR* interquartile range, *mmHg* millimetres of mercury, *mRS* modified Rankin Scale, *NIHSS* National Institutes of Health Stroke Scale, *OCSP* Oxford Community Stroke Project classification, *TACS* total anterior circulation stroke. The pre-morbid mRS, as reported by patient or representative, is the functional status of the participant prior to the onset of suspected stroke symptoms

Within the mimic group, 134 (45%) participants had been randomised to GTN and 163 (55%) to sham. Demographic and baseline clinical characteristics were similar between the GTN and sham groups except that the mean SBP was lower by 7.0 mmHg [95% CI ^−^ 12.9–^−^ 1.1; *p* = 0.021] in the group randomised to GTN.

### Mimic diagnoses

The three most common mimic diagnoses of epileptic seizure (17%), migraine or primary headache disorder (17%) and functional neurological disorder (14%), together accounted for 47% of the mimic group (Additional Table B). Other neurological (16%) and cardiovascular (7%) events represented a further 25% of presentations. A final diagnosis was unavailable in 9% of mimic cases with discharge records reporting exclusion of a stroke or TIA event but no clear diagnosis. The remaining 29% of mimic events represented a wide range of diagnoses. There was no significant difference in the proportions of final diagnoses between the treatment and sham group. In addition, for 36 participants, their qualifying event was diagnosed as an infection during at least one follow-up (Additional Table C).

### Randomised treatment

Data on adherence to the trial protocol are available for 281 (95%) cases (Additional Table D). Adherence to the first randomised treatment was near complete in both GTN and sham groups (99.3% vs 100%) but overall, only 20% of participants with a stroke mimic received the first two patches. This decreased to 9% for application of all four patches. Adherence for treatment over 2 and 4 days were much lower than for stroke/TIA participants [12]. The most common reason for non-adherence in the stroke mimic group was discontinuation following an early diagnosis of non-stroke (66%). There was no difference in adherence to protocol between the GTN and sham groups. However, patients with a final diagnosis of mimic received less treatment than those with a stroke diagnosis (Additional Table E).

There were 25 protocol violations in the ambulance; these were related to the inclusion of patients beyond 4 h, low FAST score (< 2), low SBP (< 120 mmHg), resident in a nursing home, and failure to notify the hospital (Additional Table F). There were three protocol violations in hospital; two involved not administering the treatment on Day 2 and one was failure to obtain secondary consent.

### Primary clinical outcome

**Fig. 2:**
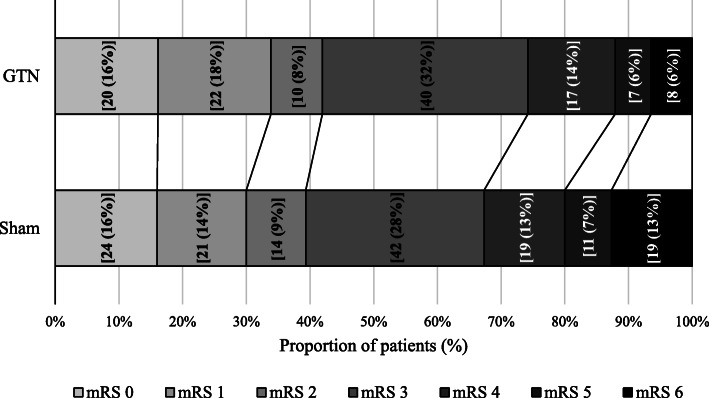
Distribution of mRS scores at day 90 for GTN versus sham among 274 stroke mimic participants. Comparison of GTN versus sham, adjusted common odds ratio 0.54 (0.34, 0.85), *p* = 0.008, by ordinal logistic regression, with adjustment for age, sex, pre-morbid modified Rankin Scale, face-arms-speech-time test, pre-treatment SBP, final diagnosis (stroke mimic) and time to randomisation. The effect of treatment for GTN versus sham is shown as adjusted common odds ratio (acOR)

**Fig. 3:**
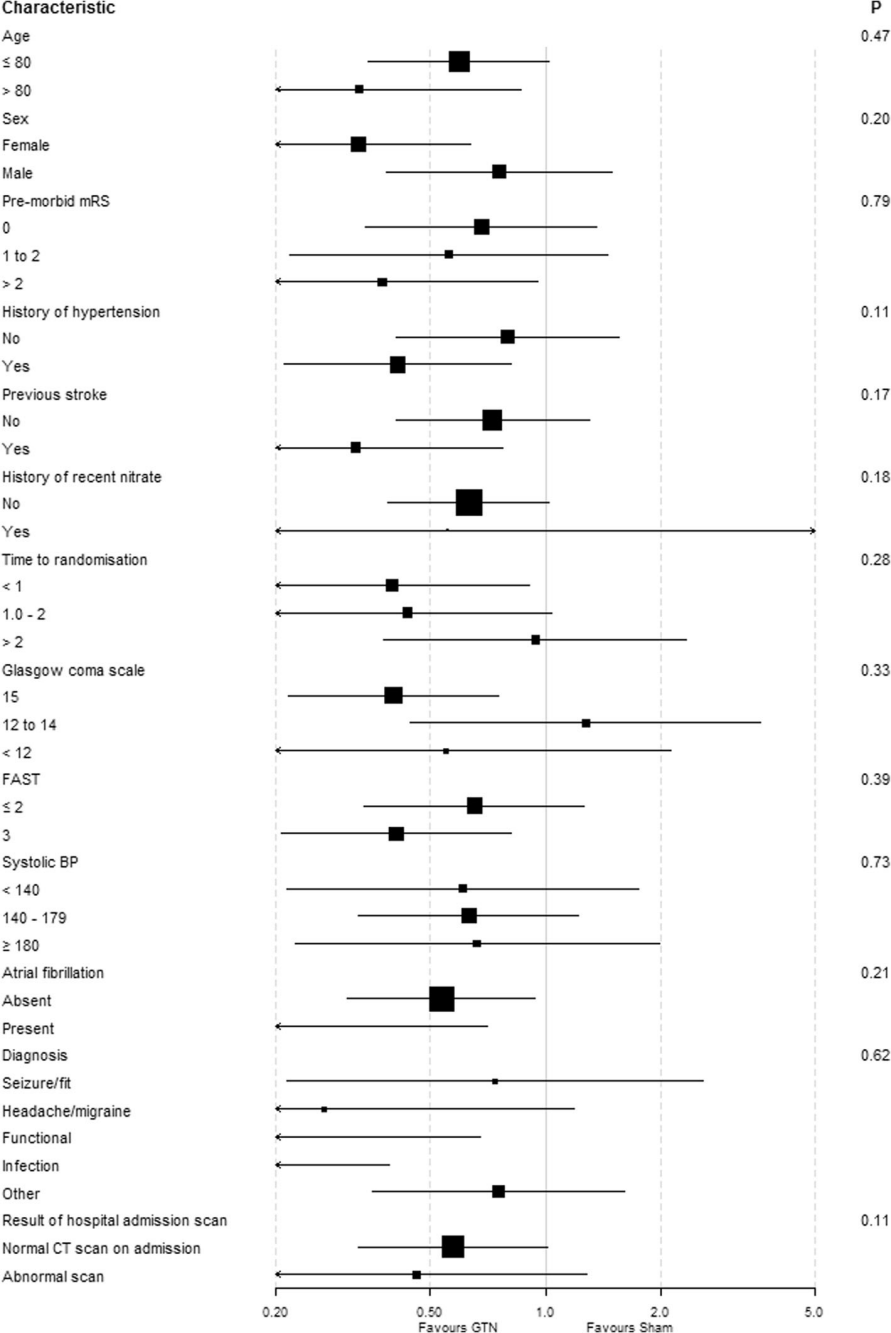
Forest plot showing modified Rankin Scale, analysed as adjusted ordinal outcome, in subgroup of participants with stroke mimics, with p-value for interaction. Heterogeneity of the treatment effect on the primary outcome was assessed in by adding an interaction term to an ordinal logistic regression model with adjustment for age, sex, pre-morbid modified Rankin Scale (mRS), face-arm-speech time test, pre-treatment systolic blood pressure (SBP), final diagnosis (stroke mimic) and time to randomisation

The primary outcome (mRS score) was measured at 90 days in 274 (92%) participants in the mimic group. A minority of participants refused or were lost to follow-up. Participants randomised to sham treatment had a median mRS of 3 [1, 4] at 90 days (Table 2). Among participants with a mimic, GTN was associated with reduced likelihood of poor 90-day mRS score, compared to sham treatment: odds ratio 0.54 (95% CI 0.34–0.85; *p* = 0.008) (Fig. 2). In a post hoc analysis, this finding was also observed when death was excluded, i.e. mRS 0–5 (OR: 0.55 (0.34, 0.91), *p* = 0.019, *N* = 247). When considering the primary outcome, no differences were found between GTN versus sham in any subgroup of participants with a stroke mimic (Fig. 3). In a further post hoc analysis, the positive effect of GTN was not localised to any particular type of mimic (Fig. 3) or other post hoc subgroups (Additional Table B).

**Table 2:** Primary and main secondary outcomes at days 4 and 90 in participants diagnosed with a stroke mimic. Data are number (%), median [IQR], or mean (standard deviation)

	N	GTN	Sham	aOR/aMD (95% CI), adjusted	***p***-value
**Day 90 mRS (/6)**					
All	274	3 [1,4]	3 [1,4]	0.5 (0.3, 0.9)	**0.008**
**Sensitivity analyses**					
Per-protocol	245	3 [1,4]	3 [1,4]	0.6 (0.4, 0.9)	**0.026**
With multiple imputation	297	3 [1,4]	3 [1,4]	0.6 (0.4, 0.9)	**0.013**
mean mRS	274	2.5 (1.7)	2.8 (1.9)	−0.5 (− 0.8, − 0.1)	**0.012**
mRS, unadjusted	274	3 [1,4]	3 [1,4]	0.8 (0.5, 1.2)	0.27
mRS > 2 (%)	274	72 (58.1)	91 (60.7)	0.6 (0.3, 1.2)	0.18
mRS > 2, unadjusted (%)	274	72 (58.1)	91 (60.7)	0.9 (0.6, 1.5)	0.66
mRS, Received thrombolysis	8	1 [1,2]	1 [0,1]	–	–
mRS, No thrombolysis	266	3 [1,4]	3 [1,4]	0.6 (0.4, 0.9)	**0.013**
**Admission**					
NIHSS (/42)	176	5.7 (6.2)	5.3 (5.3)	−0.2 (−1.8, 1.4)	0.82
FAST (hospital admission) [/3]	186	1.4 (1.1)	1.5 (1.0)	−0.2 (− 0.5, 0.1)	0.19
OCSP, TACS (%)	224	15 (15.8)	25 (19.4)	0.8 (0.4, 1.7)	0.57
GCS admission	241	14.4 (1.4)	14.2 (1.8)	0.3 (−0.1, 0.7)	0.13
**Day 4 (or discharge)**					
Death (%)	279	2 (1.6)	1 (0.6)	2.2 (0.2, 29.8)	0.56
Patients with an SAE (%)	279	6 (4.8)	10 (6.5)	0.8 (0.3, 2.5)	0.73
Infection (%)	275	11 (8.9)	17 (11.3)	0.5 (0.2, 1.2)	0.13
Neurological deterioration (%)	52	2 (7.7)	3 (11.5)	0.1 (0.0, 10.4)	0.27
Neurological deterioration, clinical (%)	275	6 (4.8)	4 (2.6)	2.3 (0.6, 9.4)	0.25
Headache (%)	274	8 (6.5)	8 (5.3)	1.4 (0.5, 4.1)	0.54
Hypotension, SBP < 90 mmHg (%)	274	3 (2.4)	0 (0)	–	–
Hypertension, SBP > 180 mmHg (%)	274	17 (13.8)	15 (9.9)	2.1 (0.9, 4.9)	0.090
Feeding: non-oral (%)	243	7 (6.4)	7 (5.3)	1.1 (0.4, 3.5)	0.87
Glasgow coma scale (/15)	114	14.2 (2.5)	14.2 (2.7)	0.1 (−0.9, 1.1)	0.79
NIHSS (/43)	57	4.5 (10.9)	4.9 (9.7)	−0.6 (−6.4, 5.3)	0.85
**Hospital events**					
Length of stay (days)	279	4.8 (8.5)	5 (6.9)	−0.6 (−2.4, 1.1)	0.48
Died in hospital (%)	279	6 (4.8)	3 (1.9)	3.7 (0.8, 17.1)	0.098
Died or discharged to institution (%)	271	13 (10.8)	19 (12.6)	0.8 (0.3, 1.7)	0.49
**Day 90**					
Death (%)	281	8 (6.3)	19 (12.3)	0.5 (0.2, 1.2)	0.11
Disposition (%)	260	1 [1,1]	1 [1,1]	0.6 (0.3, 1.2)	0.14
EQ-5D HUS (/1)	257	0.5 (0.4)	0.5 (0.4)	0.1 (0.0, 0.2)	**0.031**
Quality of life, EQ-VAS (/100)	240	57.3 (25.8)	51.9 (30.3)	6.5 (−0.2, 13.3)	0.057
Barthel Index (/100)	253	75.2 (35.1)	71.7 (39.2)	6.3 (−1.4, 14.0)	0.11
Disability, Barthel index < 60 (%)	253	27 (23.9)	33 (23.6)	0.9 (0.4, 1.9)	0.73
TICS-M	112	19.3 (10.5)	15 (11.0)	3.3 (−0.0, 6.5)	0.052
tMMSE	116	15.5 (8.0)	12.7 (9.0)	2.4 (−0.1, 4.9)	0.061
Animal naming	114	14.7 (9.5)	11.8 (10.1)	1.7 (−1.3, 4.7)	0.26
Zung Depression Scale (/100)	139	62.5 (24.3)	62.4 (27.1)	−2.3 (−9.9, 5.3)	0.55
Home time (days)	221	91 (36.2)	85.7 (39.7)	6.3 (−3.0, 15.7)	0.19
Global analysis	112	–	–	−0.1 (−0.2, 0.0)	0.15

*aMD* adjusted mean difference, *aOR* adjusted odds ratio, *CI* confidence interval, *EQ-5D HUS* EuroQol EQ-5D Health utility scores, *FAST* face-arm-speech test, *GCS* Glasgow Coma Scale, *GTN* glyceryl trinitrate, *mmHg* millimetres of mercury, *MI* multiple imputation, *mRS* modified Rankin Scale, *NIHSS* National Institutes of Health Stroke Scale, *OCSP* Oxford Community Stroke Project classification, *PP* per protocol analysis, *SAE* serious adverse event, *TACS* total anterior circulation stroke, *TICS-M* Telephone Interview for Cognitive Status -Modified, *t-MMSE* modified telephone Mini-Mental State Examination

At the Day 365 follow-up, mRS scores were measured for 279 (94%) stroke mimic patients. Those randomised to the GTN group continued to have a significantly better functional outcome than those in the sham group: OR 0.53 (95% CI 0.33–0.84; *p* = 0.007) (Additional Table G).

### Secondary outcomes

Overall, the median length of stay was 4 days [IQR 2, 8] with no significant difference between the GTN and sham groups (Table 2). The course of BP over 4 days of treatment did not reveal any sustained difference between the treatment groups (Additional Table A). There was no difference for in-hospital interventions (Additional Table H) or neuroimaging results (Additional Table I). The only significant difference between the two groups at 90 days was in the EQ-5D health utility scores (Table 2) with the group randomised to GTN scoring higher than those who received the sham treatment [aMD 0.1; 95% CI 0.0–0.2; *p* = 0.031]. However, this difference was not sustained at Day 365 (Additional Table G).

### Safety

**Fig. 4:**
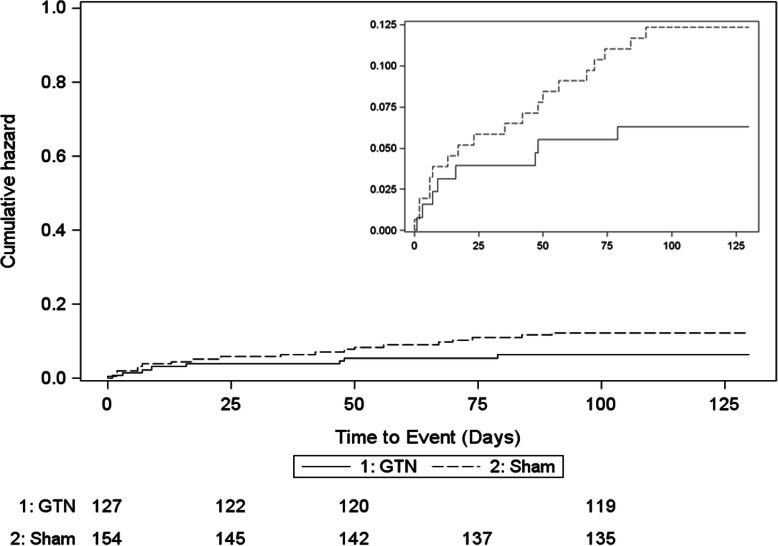
Kaplan-Meier curve for time to death in participants with a stroke mimic, by assigned treatment group. Comparison of GTN versus sham, adjusted hazard ratio 0.49 (95% confidence intervals 0.20, 1.19), *p* = 0.11, by Cox proportional hazards regression with adjustment for age, sex, pre-morbid modified Rankin Scale, face-arms-speech time test, pre-treatment SBP, final diagnosis (stroke mimic) and time to randomisation

There was no difference in the proportion of patients who died in hospital or were discharged to a residential care (Table 2, Fig. 4). The causes of death did not differ between GTN and sham (Additional Table J). Similarly, there was no difference in serious adverse events (Additional Table K).

## Discussion

### Summary of results and comparison with other studies

In our further analysis of the RIGHT-2 study, we found that 26% of the 1149 cases suspected by paramedics to be a stroke had a non-stroke final diagnosis. Patients with a stroke mimic were younger, had less atrial fibrillation, lower BP, fewer FAST positive signs, and a longer onset-to-randomisation compared to those with a confirmed stroke. The most common stroke mimics were neurological conditions; epileptic seizures, migraines and primary headache disorder, and functional neurological illness accounted for almost half of all the cases. The only significant difference between groups at baseline was a lower SBP in the GTN group. At 90 days, patients with stroke mimics had better mRS scores than those with stroke and this finding was maintained in sensitivity analyses and at 365 days. The lack of difference in the rate of serious adverse events between the two groups supports the safety of the GTN intervention among patients with stroke mimic conditions.

These findings add to prior work on prehospital stroke recognition. The rate of 26% stroke mimics is consistent with pooled results for 6870 patients in physician and paramedic-led EMS systems and larger reviews that included pre and in-hospital settings [6, 17]. Our results corroborated previous reports that stroke mimic patients are younger, less likely to display atrial arrhythmias, have a lower BP, and milder stroke signs at presentation compared to stroke patients [5, 18–21]. In contrast to earlier findings, we did not observe that mimic patients are more often female, have more vascular risk factors or a history of previous stroke [5, 18–20, 22]. The common stroke mimic conditions were similar to those seen in other studies [5, 18–20, 22].

The key but unexpected finding was that 90-day and one-year functional outcomes were better with GTN than the sham. This was despite the absence of any significant demographic or clinical differences between the two treatment groups at baseline (other than SBP), or during their in-hospital care. In addition, the 90-day quality of life score was higher in the GTN group. We suggest several possible explanations.

First, although there were no imbalances in measured prognostic factors between the groups at baseline, there may have been imbalances in unmeasured factors. Second, it is possible that cardiovascular and cerebrovascular events were missed, possibly due to atypical presentation, and included among the mimics. The subgroups with the highest odds of a better outcome with GTN were aged over 80, female, AF, hypertension, previous stroke, normal GCS, and high score on FAST. Third, it is possible that bias in the assessment of outcomes favoured GTN. However, this is unlikely due to the trial design which utilised remote assessment of outcomes at follow-up by a blinded assessor. Fourth, the greater number of deaths among mimic cases who received the sham intervention could have influenced the results. However, a comparison of the 90-day mRS scores for surviving cases was still in favour of GTN and anyway if GTN were effective, it might well reduce death as well as dependence.

Fifth, the results could have been caused by chance, particularly given the small sample size and the moderate rate of non-adherence to the study protocol in the mimic group. Stroke mimic cases in both treatment groups included a wide variety of different neurological and non-neurological diagnoses and it is difficult to explain the effect of GTN across these disorders. Further, EQ-5D differed at day 90 (with a tendency at day 365) in favour of the GTN group, and the point estimate of the BI also favoured the GTN group (although not meeting significance).

Last, the results may reflect an actual treatment effect whereby GTN improves outcome in non-stroke mimics. GTN dilates the blood vessels, increases blood supply and lowers BP due to smooth muscle relaxation. This vasodilatory effect of GTN may improve vasospastic migraine which can present as hemiparesis or hemianopia. The improvement in the seizure group could again be attributed to vasodilation by GTN. Brain oedema has been observed in patients scanned shortly after seizure activity and would cause compression of smaller vessels. Further, NO has generic antimicrobial effects (P Bath, review in preparation) and so might have attenuated the infectious causes of mimic.

This study has direct implications for pre-hospital stroke research. In particular, the frequency of stroke mimic conditions may have an unexpected impact in any intention-to-treat analysis. Mobile stroke units (where available) may still not be the solution with high rates of mimics observed among call-outs [23]. Even hospital hyperacute stroke trials are not immune to mimics with 17% of the patients enrolled into the NOR-TEST trial having a final diagnosis of a stroke mimic [24]. For now, prehospital trials will need to be designed with the impact of mimics in mind. Developing point of care diagnostics to improve accuracy in selecting the intended trial population of stroke patients is vital [25].

### Strengths and limitations

This study used high-fidelity data and the potential for bias was reduced by the limited inclusion criteria and the use of community recruitment. However, at 297 cases, the sample size was relatively small, and some cases were lost to follow-up. We also noted baseline blood pressure differences between the two study groups. Further, the use of simple randomisation may have contributed to potential undetected baseline imbalance. This approach allowed for rapid randomisation and treatment administration, but future trials could consider using phone or internet-based randomisation in the pre-hospital arena at greater expense.

## Conclusions

Close to a quarter of patients suspected by paramedics to be having an acute stroke are subsequently diagnosed with a non-stroke condition. In this study, it is unclear why administration of transdermal GTN was associated with an improvement in mRS score at 90 days and one year but is likely to represent an undetected baseline imbalance or chance. The lack of difference in the rate of serious adverse events between the two groups supported the safety of GTN intervention in this population with stroke mimic conditions. Future trials should try to improve the discrimination of stroke mimics.

## Supplementary information

**Additional file 1.** Table A. Baseline ambulance and hospital admission characteristics of Mimic versus non-Mimic patients enrolled in the RIGHT-2 trial. Data are number (%), median [IQR], or mean (standard deviation). Differences in means, medians and proportions are accompanied by 95% confidence intervals. Table B. Final diagnosis of mimics. Data are number (%). Table C. Cases whose qualifying event was described as an infection at any time-point (36 participants). Table D. Adherence and reasons for non-adherence in GTN versus sham groups. Data are number (%). Table E. Adherence and reasons for non-adherence in mimic versus non-mimic groups. Data are number (%). Table F. Protocol violations. Table G. Primary and main secondary outcomes at day 365 in all patients with a stroke mimic, except where stated. Data are number (%), median [IQR], or mean (standard deviation). Table H. In-hospital interventions. Table I. Neuroimaging on admission to hospital and day 2. Data are number (%), median [IQR], or mean (standard deviation). Table J. Causes of death among participants with a stroke mimic. Table K. Serious adverse events. Fig. A. Blood pressure curves. Fig. B. Forest plot of clinical and imaging information in participants with a stroke mimic. Fig. C. Boxplot of Day 90 mRS, by infection diagnosis (27 participants – one participant with infection is missing their mRS score).

## Data Availability

Individual participant data will be shared with the Virtual International Stroke Trials Archive (VISTA) collaboration. From Jan 1, 2022, the Chief Investigator and Trial Steering Committee will consider other requests to share individual participant data via email at: right-2@nottingham.ac.uk. We will require a protocol detailing hypothesis, aims, analyses, and intended tables and figures. Where possible, we will perform the analyses; if not, de-identified data and a data dictionary will be supplied for the necessary variables for remote analysis. Any sharing will be subject to a signed data access agreement. Ultimately, the data will be published.

## Abbreviations

BI: Barthel index
BP: Blood pressure
CI: Confidence interval
FAST: Face-Arm-Speech-Time
EQ: European Quality of Life
EQ-5D-3L: European Quality of Life-5 dimensions-3 level
ENOS: Efficacy of Nitric Oxide in Stroke trial
GCS: Glasgow coma scale
GTN: Glyceryl trinitrate
IQR: Inter-quartile range
HSUV: Health status utility value
mmHg: Millimetres of mercury
mRS: Modified Rankin scale
OR: Odds ratio
RIGHT-2: Rapid Intervention with Glyceryl trinitrate in Hypertensive stroke Trial-2
SBP: Systolic blood pressure
SD: Standard deviation
TIA: Transient ischaemic attack
TICS-M: Telephone Interview for Cognition Scale-modified
UK: United Kingdom
ZDS: Zung depression score

## References

[1] Leonardi-Bee J, Bath PM, Phillips SJ, Sandercock PAG, IST Collaborative Group (2002). Blood pressure and clinical outcomes in the International Stroke Trial. Stroke.

[2] National Guideline Centre (2019). Stroke and transient ischaemic attack in over 16s: diagnosis and initial management.

[3] Fernandes PM, Whiteley WN, Hart SR, Salman RA (2013). Strokes: Mimics and chameleons. Pract Neurol.

[4] Hand PJ, Kwan J, Lindley RI, Dennis MS, Wardlaw JM (2006). Distinguishing between stroke and mimic at the bedside: the brain attack study. Stroke.

[5] Ali SF, Viswanathan A, Singhal AB, Rost NS, Forducey PG, Davis LW (2014). The TeleStroke mimic (TM)-score: a prediction rule for identifying stroke mimics evaluated in a telestroke network. J Am Heart Assoc.

[6] McClelland G, Rodgers H, Flynn D, Price CI (2019). The frequency, characteristics and aetiology of stroke mimic presentations: a narrative review. Eur J Emerg Med.

[7] Harbison J, Hossain O, Jenkinson D, Davis J, Louw SJ, Ford GA (2003). Diagnostic accuracy of stroke referrals from primary care, emergency room physicians, and ambulance staff using the face arm speech test. Stroke.

[8] Kothari R, Hall K, Brott T, Broderick J (1997). Early stroke recognition: developing an out-of-hospital NIH Stroke Scale. Acad Emerg Med.

[9] Gropen TI, Gokaldas R, Poleshuck R, Spencer J, Janjua N, Szarek M (2014). Factors related to the sensitivity of emergency medical service impression of stroke. Prehosp Emerg Care.

[10] Bath PM, Scutt P, Anderson CS, Appleton JP, Berge E, Cala L (2019). Prehospital transdermal glyceryl trinitrate in patients with ultra-acute presumed stroke (RIGHT-2): an ambulance-based, randomised, sham-controlled, blinded, phase 3 trial. Lancet.

[11] Appleton JP, Scutt P, Dixon M, Howard H, Haywood L, Havard D (2019). Ambulance-delivered transdermal glyceryl trinitrate versus sham for ultra-acute stroke: Rationale, design and protocol for the Rapid Intervention with Glyceryl trinitrate in Hypertensive stroke Trial-2 (RIGHT-2) trial (ISRCTN26986053). Int J Stroke.

[12] The RIGHT-2 Investigators (2019). Supplementary appendix to: Prehospital transdermal glyceryl trinitrate in patients with ultra-acute presumed stroke (RIGHT-2): an ambulance-based, randomised, sham-controlled, blinded, phase 3 trial.

[13] Lees KR, Bath PM, Schellinger PD, Kerr DM, Fulton R, Hacke W (2012). Contemporary outcome measures in acute stroke research: choice of primary outcome measure. Stroke.

[14] The ENOS Trial Investigators (2015). Efficacy of nitric oxide, with or without continuing antihypertensive treatment, for management of high blood pressure in acute stroke (ENOS): a partial-factorial randomised controlled trial. Lancet.

[15] Scutt P, Appleton JP, Dixon M, Woodhouse LJ, Sprigg N, Wardlaw JM (2018). Statistical analysis plan for the ‘Rapid Intervention with Glyceryl trinitrate in Hypertensive stroke Trial-2 (RIGHT-2)’. Eur Stroke J.

[16] Lachin JM (2014). Applications of the Wei-Lachin multivariate one-sided test for multiple outcomes on possibly different scales. PLoS One.

[17] Gibson LM, Whiteley WN (2013). The differential diagnosis of suspected stroke: a systematic review. J R Coll Physicians Edinb.

[18] Ali SF, Hubert GJ, Switzer JA, Majersik JJ, Backhaus R, Shepard LW (2018). Validating the TeleStroke mimic score: a prediction rule for identifying stroke mimics evaluated over telestroke networks. Stroke.

[19] Kneihsl M, Enzinger C, Niederkorn K, Wünsch G, Müller L, Culea V, et al. Stroke referrals from nursing homes: high rate of mimics and late presentation. Cerebrovasc Dis. 2018;45(3-4):109–14. 10.1159/000487813. 10.1159/000487813 29539602

[20] Kvistad CE, Novotny V, Naess H, Hagberg G, Ihle-Hansen H, Waje-Andreassen U (2019). Safety and predictors of stroke mimics in The Norwegian Tenecteplase Stroke Trial (NOR-TEST). Int J Stroke.

[21] Natteru P, Mohebbi MR, George P, Wisco D, Gebel J, Newey CR. Variables that best differentiate in-patient acute stroke from stroke-mimics with acute neurological deficits. Stroke Res Treat. 2016;2016:1-6. 10.1155/2016/4393127. 10.1155/2016/4393127 PMC5168479 28050311

[22] Tobin WO, Hentz JG, Bobrow BJ, Demaerschalk BM. Identification of stroke mimics in the emergency department setting. J Brain Dis. 2009;1:19–22. 10.4137/jcnsd.s2280. 10.4137/jcnsd.s2280 PMC3676321 23818805

[23] Ebinger M, Winter B, Wendt M, Weber JE, Waldschmidt C, Rozanski M (2014). Effect of the use of ambulance-based thrombolysis on time to thrombolysis in acute ischemic stroke: a randomized clinical trial. JAMA.

[24] Logallo N, Novotny V, Assmus J, Kvistad CE, Alteheld L, Rønning OM (2017). Tenecteplase versus alteplase for management of acute ischaemic stroke (NOR-TEST): a phase 3, randomised, open-label, blinded endpoint trial. Lancet Neurol.

[25] Lumley HA, Flynn D, Shaw L, McClelland G, Ford GA, White PM (2020). A scoping review of pre-hospital technology to assist ambulance personnel with patient diagnosis or stratification during the emergency assessment of suspected stroke. BMC Emerg Med.

