# Supplementary material for: Pre-hospital transdermal glyceryl trinitrate in patients with stroke mimics: data from the RIGHT-2 randomised-controlled ambulance trial
Source: BMC Emerg Med. 2022 Jan 10;22:2. doi: 10.1186/s12873-021-00560-x (PMC8744321; doi:10.1186/s12873-021-00560-x)
Supplement: Supplementary file 1 — Additional file 1. Table A. Baseline ambulance and hospital admission characteristics of Mimic versus non-Mimic patients enrolled in the RIGHT-2 trial. Data are number (%), median [IQR], or mean (standard deviation). Differences in means, medians and proportions are accompanied by 95% confidence intervals. Table B. Final diagnosis of mimics. Data are number (%). Table C. Cases whose qualifying event was described as an infection at any time-point (36 participants). Table D. Adherence and reasons for non-adherence in GTN versus sham groups. Data are number (%). Table E. Adherence and reasons for non-adherence in mimic versus non-mimic groups. Data are number (%). Table F. Protocol violations. Table G. Primary and main secondary outcomes at day 365 in all patients with a stroke mimic, except where stated. Data are number (%), median [IQR], or mean (standard deviation). Table H. In-hospital interventions. Table I. Neuroimaging on admission to hospital and day 2. Data are number (%), median [IQR], or mean (standard deviation). Table J. Causes of death among participants with a stroke mimic. Table K. Serious adverse events. Fig. A. Blood pressure curves. Fig. B. Forest plot of clinical and imaging information in participants with a stroke mimic. Fig. C. Boxplot of Day 90 mRS, by infection diagnosis (27 participants – one participant with infection is missing their mRS score). [file 12873_2021_560_MOESM1_ESM.pdf]

Additional Files

Additional Table A. Baseline ambulance and hospital admission characteristics of Mimic versus non-Mimic patients enrolled in the RIGHT-2 trial. Data are number (%), median [IQR], or mean (standard deviation). Differences in means, medians and proportions are accompanied by 95% confidence intervals.

	All	Mimic	Non-mimic	Difference	2p
**Ambulance data (pre-randomisation)**					
Number of patients	1149	297	852		
Age (years)	72 (15)	67 (18)	75 (13)	-7.9 (-9.8, -6.0)	**<0.001**
< 60 (%)	227 (20)	107 (36)	120 (14)		
60-70 (%)	195 (17)	45 (15)	150 (18)		
70-80 (%)	292 (25)	55 (19)	237 (28)		
>=80 (%)	435 (38)	90 (30)	345 (40)		
Sex (female) (%)	555 (48)	157 (53)	398 (57)	-6.2 (-12.7, 0.4)	0.068
Time from onset to randomisation (minutes)	71 [45,116]	75 [47,126]	70 [45,108]	7.0 (1.0, 13.0)	**0.022**
ECG, AF/flutter (%)	187 (20)	29 (13)	158 (23)	9.9 (4.6, 15.3)	**0.0013**
Systolic blood pressure (mmHg)	162 (25)	159 (26)	163 (25)	-4.2 (-7.5, -0.9)	**0.014**
Diastolic blood pressure (mmHg)	92 (18)	91 (16)	92 (18)	-1.0 (-3.4, 1.4)	0.42
Heart rate (bpm)	82 (19)	83 (19)	82 (19)	1.0 (-1.4, 3.5)	0.41
Glasgow coma scale	14 (2)	14 (2)	14 (2)	-0.1 (-0.3, 0.2)	0.68
Glasgow coma scale <14 (%)	302 (26)	73 (25)	229 (27)	2.1 (-3.7, 7.9)	0.49
FAST score (/3)	3 (1)	2 (1)	3 (0)	-0.2 (-0.2, -0.1)	**<0.001**
FAST score =3 (%)	690 (60)	144 (49)	546 (64)	15.3 (8.7, 21.8)	**<0.001**
**Hospital admission data (post randomisation)**					
Number of patients with data	297	134	163		
Ethnic group, non-white (%)	35 (13)	15 (12)	20 (13)	1.2 (-6.7, 9.0)	0.77
Ethnicity, White (%)	242 (87)	110 (88)	132 (87)		
Ethnicity, Black (%)	14 (5)	5 (4)	9 (6)		
Ethnicity, Asian (%)	18 (6)	10 (8)	8 (5)		
Ethnicity, Other (%)	3 (1)	0 (0)	3 (2)		
Pre-morbid mRS >2 (%)	79 (28)	39 (31)	40 (26)	-4.7 (-15.3, 5.9)	0.38
Medical history (%)					
Hypertension	142 (51)	61 (49)	81 (53)	4.1 (-7.8, 15.9)	0.50
Diabetes mellitus	59 (21)	27 (22)	32 (21)	-0.6 (-10.3, 9.2)	0.91
Previous stroke	85 (31)	37 (30)	48 (32)	2.2 (-8.7, 13.1)	0.70
Ischaemic heart disease	58 (21)	29 (24)	29 (19)	-4.2 (-14.1, 5.6)	0.39
Smoking, current	54 (24)	26 (25)	28 (23)	-2.2 (-13.4, 8.9)	0.69

	All	Mimic	Non-mimic	Difference	2p
Antiplatelets	69 (36)	36 (40)	33 (33)	-7.0 (-20.7, 6.7)	0.32
Anticoagulants	33 (17)	15 (17)	18 (18)	1.0 (-9.7, 11.7)	0.86
Either	96 (51)	47 (52)	49 (49)	-3.2 (-17.5, 11.0)	0.66
OCSP syndrome, TACS (%)	40 (18)	15 (16)	25 (19)	3.6 (-6.4, 13.6)	0.49
NIHSS (/43)	4 [2,8]	4 [1,9]	4 [2,7]	0.0 (-1.0, 1.0)	0.64

Additional Table B. Final diagnosis of mimics. Data are number (%)

Mimic diagnosis	All	GTN	Sham
Number of participants	297	134	163
Neurological	*187 (63.0)*	*83 (61.9)*	*104 (63.8)*
Epileptic seizure	50 (16.8)	20 (14.9)	30 (18.4)
Migraine/headache	49 (16.5)	26 (19.4)	23 (14.1)
Functional neurological illness	41 (13.8)	19 (14.2)	22 (13.5)
Other [a]	10 (3.4)	6 (4.5)	4 (2.5)
Neuropathy	9 (3.0)	2 (1.5)	7 (4.3)
Previous stroke	6 (2.0)	1 (0.7)	5 (3.1)
Subdural haemorrhage	6 (2.0)	1 (0.7)	5 (3.1)
Acute vestibular syndrome	5 (1.7)	4 (3.0)	1 (0.6)
Subarachnoid haemorrhage	3 (1.0)	1 (0.7)	2 (1.2)
Dementia	3 (1.0)	0 (0.0)	3 (1.8)
Traumatic brain injury	2 (0.7)	2 (1.5)	0 (0.0)
Transient global amnesia (TGA)	1 (0.3)	0 (0.0)	1 (0.6)
Encephalitis	1 (0.3)	1 (0.7)	0 (0.0)
Carotid artery dissection	1 (0.3)	0 (0.0)	1 (0.6)
Diagnosis unknown [b]	*27 (9.1)*	*11 (8.2)*	*16 (9.8)*
Cardiovascular	*21 (7.1)*	*8 (6.0)*	*13 (8.0)*
Syncope	15 (5.1)		
Other [c]	6 (2.0)		
Renal [d]	*12 (4.0)*	*8 (6.0)*	*4 (2.5)*
Respiratory [e]	*12 (4.0)*	*5 (3.7)*	*7 (4.3)*
Malignancy [f]	*12 (4.0)*	*6 (4.5)*	*6 (3.7)*
Metabolic	*10 (3.4)*	*7 (5.2)*	*3 (1.8)*
Alcohol excess	4 (1.3)		
Hypoglycaemia	3 (1.0)		
Hyperglycaemia	1 (0.3)		
Other [g]	2 (0.7)		
Infection, other non-CNS [h]	*4 (1.3)*	*0 (0.0)*	*4 (2.5)*
Gastrointestinal [i]	*4 (1.3)*	*3 (2.2)*	*1 (0.6)*
Fall	*4 (1.3)*	*2 (1.5)*	*2 (1.2)*
Other diagnoses [j]	*3 (1.0)*	*0 (0.0)*	*3 (1.8)*
Musculoskeletal	*1 (0.3)*	*1 (0.7)*	*0 (0.0)*

[a] Neurological other: Acute focal dystonia, myoclonic jerk/hemifacial spasm, bilateral leg weakness, unknown, ataxia, central nervous system lupus, Parkinson's, possible TIA, atypical sensory symptoms, cerebral amyloid angiopathy

[b] Diagnosis unknown: non-stroke, unsure, unwell, self-discharge, no clear diagnosis

[c] Cardiovascular other: Dysrhythmia, pulmonary emboli (x2), heart failure, myocardial infarction, Takotsubo cardiomyopathy
[d] Renal: Acute kidney injury, urinary tract infection (x11)
[e] Respiratory: Pleural effusion, respiratory tract infection (x11)
[f] Malignancy: Squamous cell carcinoma with metastases, no primary site identified (x11)
[g] Metabolic other: non-alcoholic fatty liver disease, gout
[h] Infection other: sepsis unknown cause (x4). 23 infections distributed in other groups
[i] Gastrointestinal: Abdominal pain (x2), constipation, gastroenteritis
[j] Other diagnoses: anaphylaxis, ptosis, Mediterranean familial fever

Additional Table C. Cases whose qualifying event was described as an infection at any time-point (36 participants)

**Diagnosis**	**GTN**	**Sham**
All infection	18	18
Encephalitis	1 (5.6%)	1 (5.6%)
Gastrointestinal	0 (0.0%)	1 (5.6%)
Infection, other non-CNS	3 (16.7%)	5 (27.8%)
Renal	9 (50.0%)	4 (22.2%)
Respiratory	5 (27.8%)	7 (38.9%)

Additional Table D. Adherence and reasons for non-adherence in GTN versus sham groups. Data are number (%)

	All	GTN	Sham	p-value
**Patients with data**	**297**	**134**	**163**	
**Adherence (%)**				
Received all 4 days treatment	26 (8.8)	13 (9.7)	13 (8)	0.60
Received at least first 2 days of treatment	60 (20.2)	24 (17.9)	36 (22.1)	0.37
Received first treatment	296 (99.7)	133 (99.3)	163 (100)	0.27
Received one or more patches	296 (99.7)	133 (99.3)	163 (100)	0.27
Did not receive any randomised treatment	1 (0.3)	1 (0.7)	0 (0)	0.27
**Reasons for non-adherence (%)**				
Early non-stroke diagnosis given	197 (66.3)	93 (69.4)	104 (63.8)	0.31
Discharged before patch could be given	8 (2.7)	3 (2.2)	5 (3.1)	0.66
Participant/proxy refused patch	3 (1)	1 (0.7)	2 (1.2)	0.68
Doctor did not want patch administered	2 (0.7)	1 (0.7)	1 (0.6)	0.89
Procedural error	1 (0.3)	0 (0)	1 (0.6)	0.36
Died	1 (0.3)	0 (0)	1 (0.6)	0.36
Other reason	5 (1.7)	3 (2.2)	2 (1.2)	0.50
Open label GTN	15 (5.1)	5 (3.7)	10 (6.1)	0.35

Additional Table E. Adherence and reasons for non-adherence in mimic versus non-mimic groups. Data are number (%)

	All	Mimic	Stroke	p-value
**Patients with data**	**1149**	**297**	**852**	
**Adherence (%)**				
Received all 4 days treatment	408 (35.5)	26 (8.8)	382 (44.8)	<0.0001
Received at least first 2 days of treatment	631 (54.9)	60 (20.2)	571 (67.0)	<0.0001
Received first treatment	1145 (99.7)	296 (99.7)	849 (99.7)	0.97
Received one or more patches	1145 (99.7)	296 (99.7)	849 (99.7)	0.97
Did not receive any randomised treatment	4 (0.4)	1 (0.3)	3 (0.4)	0.97
**Reasons for non-adherence (%)**				
Early non-stroke diagnosis given	256 (22.3)	197 (66.3)	59 (6.9)	<0.0001
Serious adverse event	4 (0.4)	0 (0)	4 (0.5)	0.24
Adverse event (not an SAE)	2 (0.2)	0 (0)	2 (0.2)	0.40
Discharged before patch could be given	46 (4)	8 (2.7)	38 (4.5)	0.18
Participant/proxy refused patch	8 (0.7)	3 (1.0)	5 (0.6)	0.45
Doctor did not want patch administered	51 (4.4)	2 (0.7)	49 (5.8)	0.0003
Procedural error	46 (4)	1 (0.3)	45 (5.3)	0.0002
Trial medication missing / not available	20 (1.7)	0 (0)	20 (2.4)	0.0077
Died	17 (1.5)	1 (0.3)	16 (1.9)	0.058
Other reason	41 (3.6)	5 (1.7)	36 (4.2)	0.042
Open label GTN	94 (8.2)	15 (5.1)	79 (9.3)	0.022

Additional Table F. Protocol violations

Violation	All	GTN	Sham
Total number of patients with violations	32	15	17
Total number of violations	35	16	19
--- Ambulance ---			
Failure to obtain consent	0 (0.0)	0 (0.0)	0 (0.0)
No witnessed signature for proxy consent	0 (0.0)	0 (0.0)	0 (0.0)
Randomisation over 4 hours from onset of symptoms	6 (17.1)	2 (12.5)	4 (21.1)
FAST score of 0 or 1	5 (14.3)	3 (18.8)	2 (10.5)
Systolic blood pressure less than 120 mmHg	3 (8.6)	2 (12.5)	1 (5.3)
Glasgow Coma Scale less than 8	0 (0.0)	0 (0.0)	0 (0.0)
Glucose less than 2.5 mmol/L	0 (0.0)	0 (0.0)	0 (0.0)
Patient from nursing home	2 (5.7)	2 (12.5)	0 (0.0)
Patient did not receive first GTN/sham patch	0 (0.0)	0 (0.0)	0 (0.0)
Already in another trial	0 (0.0)	0 (0.0)	0 (0.0)
Hospital not informed of trial participant	0 (0.0)	0 (0.0)	0 (0.0)
No message left (notification of new participant)	9 (25.7)	4 (25.0)	5 (26.3)
--- Hospital ---			
Failure to enter ambulance baseline data sheet	0 (0.0)	0 (0.0)	0 (0.0)
Failure to enter hospital admission form	0 (0.0)	0 (0.0)	0 (0.0)
Participant did not receive second GTN/sham patch	2 (5.7)	1 (6.3)	1 (5.3)
Failure to obtain consent where applicable	1 (2.9)	0 (0.0)	1 (5.3)
Failure to enter day 4 follow-up form	0 (0.0)	0 (0.0)	0 (0.0)
Failure to enter discharge/death form	0 (0.0)	0 (0.0)	0 (0.0)
Subsequent randomisation into another trial	0 (0.0)	0 (0.0)	0 (0.0)
Failure to report/submit SAEs	0 (0.0)	0 (0.0)	0 (0.0)
--- Long-term follow-up ---			
Day 90 follow-up performed before day 83 or after day 104	2 (5.7)	0 (0.0)	2 (10.5)
Day 365 follow-up performed before day 345 or after day 385	0 (0.0)	0 (0.0)	0 (0.0)
--- Miscellaneous ---			
Any other violation of the trial protocol	5 (14.3)	2 (12.5)	3 (15.8)

Additional Table G. Primary and main secondary outcomes at day 365 in all patients with a stroke mimic, except where stated. Data are number (%), median [IQR], or mean (standard deviation).

	N	GTN	Sham	OR/MD (95% CI), adjusted	p-value
**Day 365 mRS (/6)**					
Primary outcome: All (mITT)	279	3 [0,4]	3 [1,5]	0.53 (0.33, 0.84)	**0.007**
Primary outcome: unadjusted	279	3 [0,4]	3 [1,5]	0.73 (0.48, 1.11)	0.14
**Sensitivity analyses**					
Primary outcome: all MI	297	3 [0,4]	3 [1,4]	0.55 (0.35, 0.86)	**0.0089**
Primary outcome: All (PP)	250	3 [0,4]	3 [1,5]	0.6 (0.37, 0.98)	**0.040**
Primary outcome: mean mRS	279	2.5 (2.1)	2.9 (2.1)	-0.57 (-0.96, -0.18)	**0.0038**
Primary: mRS > 2 (%)	279	68 (54)	95 (62)	0.45 (0.24, 0.87)	**0.018**
**Day 365**					
Death (%): all	288	20 (16)	30 (19)	0.63 (0.34, 1.16)	0.14
Disposition (%)	252	1 [1,1]	1 [1,2]	0.72 (0.36, 1.44)	0.35
EQ-5D HUS (/1)	246	0.4 (0.4)	0.4 (0.4)	0.04 (-0.05, 0.14)	0.37
Barthel Index (/100)	246	67.4 (42.1)	63.8 (43.4)	5.27 (-3.61, 14.16)	0.24
TICS-M	125	14.7 (12.5)	11.8 (12.2)	2.55 (-0.67, 5.76)	0.12
Zung Depression Scale (/100)	148	68.7 (28.3)	71.6 (28.2)	0.22 (-7.31, 7.75)	0.95
Global analysis	125	-	-	-0.09 (-0.22, 0.05)	0.21

Additional Table H. In-hospital interventions

	N	GTN	Sham	OR/MD (95% CI), adjusted	p-value
Intravenous alteplase (%)	281	3 (2.4)	7 (4.5)	0.47 (0.11, 2.09)	0.32
Door to needle time	10	57 [45, 79]	56 [45, 102]	-34 (-116, 48)	1.00
Other surgery (%)	274	1 (0.8)	2 (1.3)	1.18 (0.08, 17.67)	0.90
Admission to neurosurgical unit	277	1 (0.8)	3 (2.0)	0.19 (0.01, 2.88)	0.23
Admission to intensive care unit	275	2 (1.6)	5 (3.3)	0.58 (0.10, 3.31)	0.54
Artificial ventilation used	275	1 (0.8)	5 (3.3)	0.27 (0.03, 2.52)	0.25
Admission to stroke unit	279	44 (35.2)	52 (33.8)	1.00 (0.59, 1.67)	0.99
Admission to stroke rehabilitation unit	279	10 (8.0)	13 (8.4)	0.82 (0.34, 2.01)	0.67
Physiotherapy	271	51 (41.8)	59 (39.6)	0.97 (0.57, 1.65)	0.90
Occupational therapy	271	37 (30.3)	47 (31.5)	0.71 (0.40, 1.29)	0.26
Speech therapy	273	26 (21.1)	22 (14.7)	1.48 (0.75, 2.93)	0.26

Additional Table I. Neuroimaging on admission to hospital and day 2. Data are number (%), median [IQR], or mean (standard deviation).

	N	GTN	Sham	OR/MD (95% CI), adjusted	P
Normal Scan	250	10 (9.1)	12 (8.6)	1.07 (0.44, 2.57)	0.89
Onset to scan (hours)	246	2.4 [1.7,3.2]	2.7 [1.9,3.9]	-	-
Atrophy	228	97 (97.0)	120 (93.8)	2.16 (0.56, 8.35)	0.27
PVL	226	37 (37.0)	55 (43.7)	0.76 (0.44, 1.30)	0.31
Old strokes	228	69 (69.0)	84 (65.6)	1.17 (0.67, 2.04)	0.59
Extradural (%)	250	0 (0.0)	0 (0.0)	-	-
Subdural (%)	250	0 (0.0)	3 (2.1)	-	-
Atrophy	228	97 (97.0)	120 (93.8)	2.16 (0.56, 8.35)	0.27
PVL	226	37 (37.0)	55 (43.7)	0.76 (0.44, 1.30)	0.31
Old strokes	228	69 (69.0)	84 (65.6)	1.17 (0.67, 2.04)	0.59
Frailty [/3]	250	2 [1,3]	2 [1,3]	0.98 (0.62, 1.54)	0.93
SVD [/2]	250	0 [0,1]	1 [0,1]	0.77 (0.48, 1.25)	0.29

Additional Table J. Causes of death among participants with a stroke mimic

Causes of death	All	GTN	Sham
Subsequent stroke	2 (0.7)	0 (0.0)	2 (1.2)
Intra-cranial bleeding	4 (1.3)	1 (0.7)	3 (1.8)
Cancer	8 (2.7)	5 (3.7)	3 (1.8)
Pneumonia	1 (0.3)	1 (0.7)	0 (0.0)
Other respiratory	1 (0.3)	0 (0.0)	1 (0.6)
Other genito-urinary	1 (0.3)	0 (0.0)	1 (0.6)
Sudden cardiac death	2 (0.7)	0 (0.0)	2 (1.2)
Unattended/Unknown	8 (2.7)	0 (0.0)	8 (4.9)
Infection	1 (0.3)	1 (0.7)	0 (0.0)

Note: These categories are calculated fields. One participant was recorded in two categories.

Additional Table K. Serious adverse events

	All SAEs			Fatal SAEs		
	**GTN**	**Sham**	**p**	**GTN**	**Sham**	**p**
Cardiovascular	1 (0.7)	4 (2.5)	0.28	0 (0)	2 (1.2)	-
Nervous system	6 (4.5)	10 (6.1)	0.53	4 (3.0)	7 (4.3)	0.55
Respiratory	3 (2.2)	2 (1.2)	0.51	2 (1.5)	1 (0.6)	0.47
Genito-urinary	0 (0)	1 (0.6)	-	0 (0)	1 (0.6)	-
Musculoskeletal / cutaneous	0 (0)	2 (1.2)	-	0 (0)	0 (0)	-
Infection	3 (2.2)	4 (2.5)	0.90	2 (1.5)	0 (0)	-
Miscellaneous	3 (2.2)	8 (4.9)	0.24	1 (0.7)	8 (4.9)	0.071
Total patients with an SAE	13 (9.7)	28 (17.2)	0.066	8 (6.0)	19 (11.7)	0.095

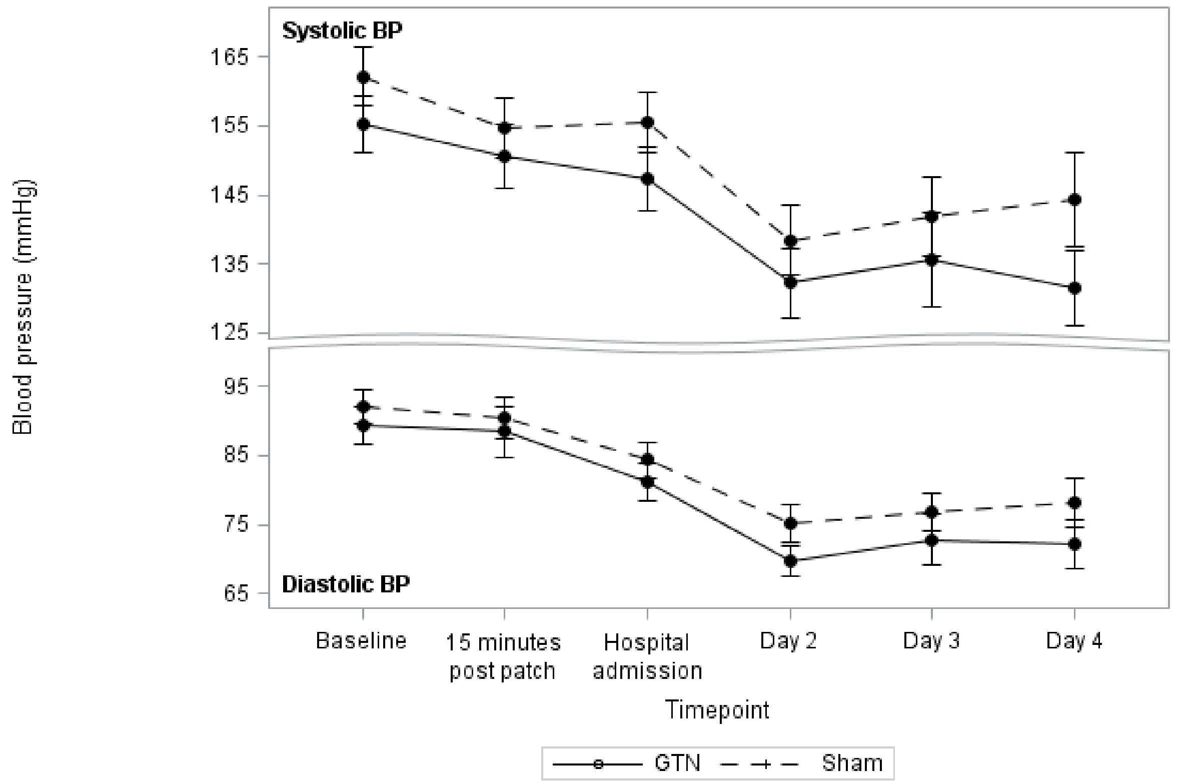

	Baseline	15 minutes post patch	Hospital admission	Day 2	Day 3	Day 4
N (GTN / Sham)	134 / 161	125 / 153	123 / 149	73 / 92	52 / 66	41 / 52
Difference (SBP / DBP)		-4.1 / -2.1	-8.4 / -3.2	-6.2 / -5.5	-6.2 / -4.0	-12.9 / -6.1
p-value (SBP / DBP)		0.56 / 0.87	0.24 / 0.16	0.22 / 0.013	0.87 / 0.33	0.091 / 0.21

Additional Figure A. Blood pressure curves

Characteristic	OR (95% CI)	P	Interaction P
Stroke syndrome			0.30
LACS	0.81 (0.34, 1.94)	0.64	
POCS	0.69 (0.09, 5.51)	0.73	
PACS	1.54 (0.74, 3.17)	0.25	
TACS	0.46 (0.13, 1.64)	0.23	
Stroke severity (NIHSS)			0.53
> 12	0.90 (0.15, 5.30)	0.91	
9 to 12	0.46 (0.08, 2.53)	0.37	
< 9	1.34 (0.73, 2.47)	0.34	
Feeding			0.61
Oral	0.79 (0.49, 1.25)	0.31	
Non-oral	0.55 (0.13, 2.24)	0.4	
Brain frailty factors on imaging			0.93
0	0.68 (0.16, 2.82)	0.59	
1	0.86 (0.46, 1.63)	0.65	
2	0.75 (0.35, 1.59)	0.45	
Small vessel disease on imaging			0.82
0	1.03 (0.55, 1.93)	0.93	
1	0.77 (0.35, 1.72)	0.52	
2	0.71 (0.21, 2.41)	0.58	
Number of patches received			0.65
1	0.73 (0.46, 1.18)	0.2	
2 to 3	0.67 (0.19, 2.33)	0.52	
4	1.52 (0.37, 6.26)	0.57	
Delta SBP			0.59
>5% decrease	0.90 (0.48, 1.67)	0.73	
-5 – 5% change	0.63 (0.28, 1.41)	0.26	
>5% increase	1.22 (0.46, 3.23)	0.68	
Antiplatelet			0.61
Yes	0.83 (0.35, 1.93)	0.66	
No	0.68 (0.35, 1.31)	0.25	
Anticoagulant			0.18
Yes	0.20 (0.05, 0.85)	0.03	
No	0.84 (0.48, 1.48)	0.54	
Atrophy			0.74
Yes	0.77 (0.47, 1.25)	0.3	
No	0.51 (0.03, 9.94)	0.66	
PVL			0.73
Yes	0.79 (0.37, 1.68)	0.54	
No	0.94 (0.50, 1.74)	0.84	
Old stroke			0.66
Yes	0.79 (0.44, 1.41)	0.43	
No	0.65 (0.28, 1.54)	0.33	

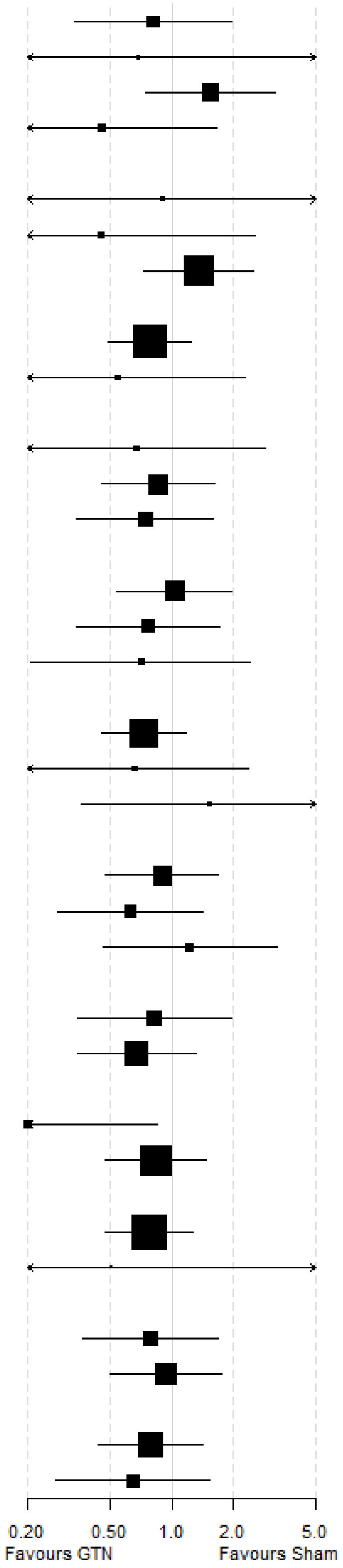

Additional Figure B. Forest plot of clinical and imaging information in participants with a stroke mimic

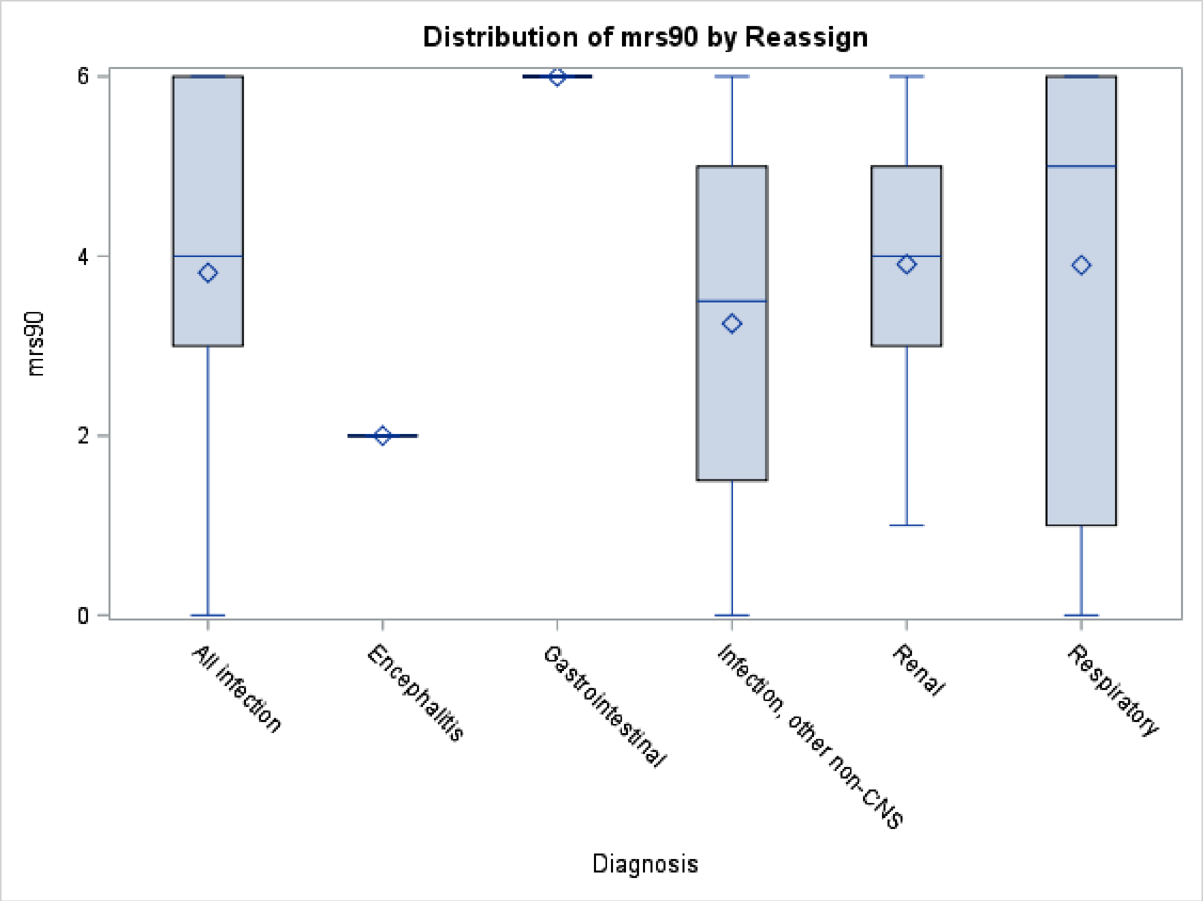

Additional Figure C. Boxplot of Day 90 mRS, by infection diagnosis (27 participants – one participant with infection is missing their mRS score)
